# Immune Checkpoint Inhibition Perturbs Neuro-immune Homeostasis and Impairs Cognitive Function

**DOI:** 10.21203/rs.3.rs-6389488/v1

**Published:** 2025-04-25

**Authors:** Onwodi V. Ifejeokwu, An Do, Sanad M. El Khatib, Nhu H. Ho, Angel Zavala, Shivashankar Othy, Munjal M. Acharya

**Affiliations:** University of California Irvine; University of California Irvine; University of California Irvine; University of California Irvine; University of California Irvine; University of California Irvine; University of California Irvine

**Keywords:** Immune checkpoint inhibition, anti-PD-1, anti-CTLA-4, melanoma, lymphocyte, brain, microglia, neuroinflammation, synaptic loss, myelin, cognitive function

## Abstract

**Background.:**

Blockade of Cytotoxic T-lymphocyte-associated protein 4 (CTLA-4) and Programmed Cell Death Protein 1 (PD-1) significantly improves progression-free survival of individuals with cancers, including melanoma. In addition to unleashing antitumor immunity, immune checkpoint inhibition (ICI) therapies disrupt immune regulatory networks critical for maintaining homeostasis in various tissues, including the central nervous system (CNS). Despite growing reports of cancer- and ICI-related cognitive impairments among survivors, our understanding of the pathophysiology of ICI-related neurodegenerative effects is limited.

**Methods.:**

In this study, using a murine model of melanoma, cognitive function tests, and neuroimmunological assays, we investigate the cellular mechanisms and impact of combinatorial blockade of CTLA-4 and PD-1 on brain function. Syngeneic melanoma was induced in a C57Bl6 mouse model using D4M-3A.UV2 melanoma cells. After confirmation of tumor growth, cancer-bearing and non-cancer mice received combinatorial treatment of anti-CTLA-4 (two doses per week) and anti-PD-1 (three doses per week) for three weeks. One month after completing ICI treatment, mice were administered learning, memory, and memory consolidation cognitive function tasks. Neuroinflammation, synaptic, and myelin integrity analyses and immune cell status in the brain were conducted to analyze neuroimmunological changes post-ICI treatment.

**Results.:**

While tumor-related alterations in brain function were evident, combination ICI specifically disrupted synaptic integrity and reduced myelin levels independent of neurogenesis and neuronal plasticity in both cancer-bearing and non-cancer mice brains. Combination ICI selectively impaired hippocampal-dependent cognitive function. This is associated with two-fold increase in T cell numbers within the brain along with immune activation of myeloid cells, especially microglia. Furthermore, an experimental autoimmune encephalomyelitis model revealed that combination ICI predisposes the CNS to exacerbated autoimmunity, highlighting neuroinflammation-related, and tumor-independent, neurodegenerative sequelae of combination ICI.

**Conclusion.:**

Our results demonstrate that combinatorial blockade of CTLA-4 and PD-1 destabilizes neuroimmune-regulatory networks and activates microglia, contributing to long-term neurodegeneration and cognitive impairments. Therefore, selectively limiting microglial activation could be a potential avenue to preserve CNS functions while maintaining the therapeutic benefits of rapidly evolving ICIs and their combinations.

## Introduction

Immunotherapies have revolutionized cancer treatment and improved overall survival. Immune Checkpoint Inhibition (ICI) therapies, which target immune regulatory pathways, have significantly increased the therapeutic response of cancers and improved life expectancy, including primary and metastatic melanoma [10, 24]. Commonly used ICI include blockade of cytotoxic T-lymphocyte associated protein 4 (CTLA-4) and programmed cell death protein 1 (PD-1) pathways, alone or in combination to rejuvenate exhausted immune cells, including cytotoxic (CD8^+^) T cells to promote tumor cell killing [10, 24]. Since the first FDA approval of anti-CTLA-4 (Ipilimumab) in 2011 [29], cancer immunotherapies are rapidly evolving, over half a dozen ICI therapies are already approved, and combinations are being extensively evaluated [49, 59]. Despite the success of ICI, off-target effects and normal tissue toxicities are evident [15, 16], which affects the quality of life of cancer survivors. Although the adverse gastrointestinal tract effects are well characterized (e.g., ICI-associated Colitis), our understanding of the cellular mechanism of neurodegenerative and neurocognitive complications ICI and their combination is largely incomplete.

Cancer survivors who underwent ICI therapy report headache, fatigue, fever, and loss of appetite [54]. Emerging clinical data suggest both short-term and long-term adverse effects of ICI on brain function [9, 23, 54]. ICI-induced short-term neurotoxicity is commonly reported in patients with melanoma, small cell lung cancer (SCLC), non-small cell lung cancer (NSCLC), and Merkel-cell carcinoma at five weeks post-therapy [17]. Long-term neurocognitive complications after ICI treatment (11 months up to 2 years) are reported in upto 41% of the melanoma survivors [46, 47]. Longitudinal studies assessing the cognitive function and psychosocial impact of Ipilimumab (anti-CTLA-4) on first-generation survivors are revealing numerous psychological issues, including anxiety, depression, and PTSD, as well as impairment in neurocognitive function [46–48]. Most observational studies on ICI-treated cancer survivors are single-arm, making it difficult to distinguish the impact of cancer burden from ICI treatment-related impact on cognition. A better understanding of the mechanisms of neurodegenerative and neurocognitive complications post-ICI treatment is a prerequisite to improve the quality of life of the increasing population of cancer survivors.

A combination of anti-CTLA-4 and anti-PD-1 treatment is highly efficacious for treating melanoma [14] and is being tested in several clinical trials for various cancers [53, 62]. However, neuroinflammatory and neurodegenerative events post-ICI, particularly for dual checkpoint inhibition, remain entirely unknown. We hypothesize that cancer survivors of dual ICI (CTLA-4 and PD-1 blockade) therapy potentially develop neurocognitive complications because of the disruption of immune regulatory networks during and post-ICI. Anti-CTLA-4 and anti-PD-1 antibodies have shown minimal permeability through the blood brain barrier [26, 50]. Additionally, ICI-related neurotoxicities are often associated with demyelination, CNS autoimmune reaction, and sensorimotor polyneuropathy [17, 19, 37]. Our previous cancer therapy studies have established that microglial activation leads to neuronal and synaptic loss, culminating in cognitive dysfunction following cranial irradiation and chemotherapies [1–5, 34, 36]. Microglia constitute about ~ 12% of all CNS cell types and serve as tissue-resident immune cells, playing both reparative and damaging roles during tissue injury, infections, or neurodegenerative conditions [42].

Understanding the neurobiological underpinnings of ICI therapy-related modulation of brain functions is key to developing mitigation strategies. In this study, using the syngeneic melanoma model in immunocompetent mice and a series of cognitive function tests, we demonstrate that blockade of CTLA-4 and PD-1 impedes hippocampal-dependent cognitive function by altering the immune landscape of the brain and activating microglia, resulting in neuroinflammation-mediated neurodegeneration.

## MATERIALS AND METHODS

Detailed methods are provided in the **Supplemental Information** section.

### Animal models, Combination CTLA-4 and PD-1 blockade, tumor induction, EAE, and cognitive function analysis.

All animals used in this study were cared forasper NIH guidelines and approved by the university animal care and use committee. Adult (8–10 week) male WT mice were divided into the following groups: *i*) WT mice injected with isotype-matched control (ITC, i.p.), *ii*) WT mice with D4MUV2 tumors injected with ITC, *iii*) WT mice injected with 1mg/dose anti-CTLA-4 (3 times per week, i.p.), and 200μg/dose anti-PD1 (2 times per week, i.p.), and *iv*) WT mice with D4MUV2 tumors injected with combination ICI as above for 3 weeks. Cancer groups received bilateral injections of 5 × 10^5^ syngeneic D4MUV2 cells that were engrafted for one week before ICI or ITC treatments. Mice underwent cognitive function testing four weeks after the last dose of ICI, followed by euthanasia and tissue harvesting at indicated time points. The cognitive function tests were open field test (OFT), light-dark box test (LDB), object location memory (OLM), and fear extinction memory (FE). Details on animal experimentation, cognitive function testing protocols, and experimental autoimmune encephalomyelitis can be found in **Supplemental Information**.

### Dual-immunofluorescence staining, confocal microscopy, and 3D algorithm-based volumetric quantification.

The PFA-fixed brains from each treatment group underwent dual-immunofluorescence staining (2 sections/brain and 4 brains/group), including GFAP, IBA1, synaptophysin, PSD95, myelin basic protein (MBP), BrdU-NeuN, NeuN-cFoS, and IBA1-CD68. Imaris modules were used to blindly and unbiasedly 3D deconvolute and volumetrically quantify neuronal and glial surfaces. All representative images are from technical replicates of indicated brain regions from biological replicates. Please refer to **Supplemental Information** for details regarding antibodies and immunostaining.

### Single-cell isolation from the brain and flow cytometry.

Mice were euthanized 72 hours after the last injection for flow cytometry of the brain and cytokine analysis. FACS analysis was conducted using a Novocyte Quanteon and analyzed using NovoExpress (Agilent Technologies, California, US). Please see the Supplemental Information for details about the brain cell isolation protocol.

### Data analysis.

Data are pooled from at-least two independent experiments. Statistical analyses were performed to confirm significance (GraphPad Prism, v8.0) using two-way ANOVA or repeated measures ANOVA, and Bonferroni’s multiple comparisons test. All results are expressed as the mean values ± SEM. All analyses considered a value of P≤0.05 to be statistically significant.

### Data availability.

All data reported will be shared upon a reasonable request.

## Results

### CTLA-4 and PD-1 blockade eliminates melanoma and impairs cognitive function

**Figure 1:**
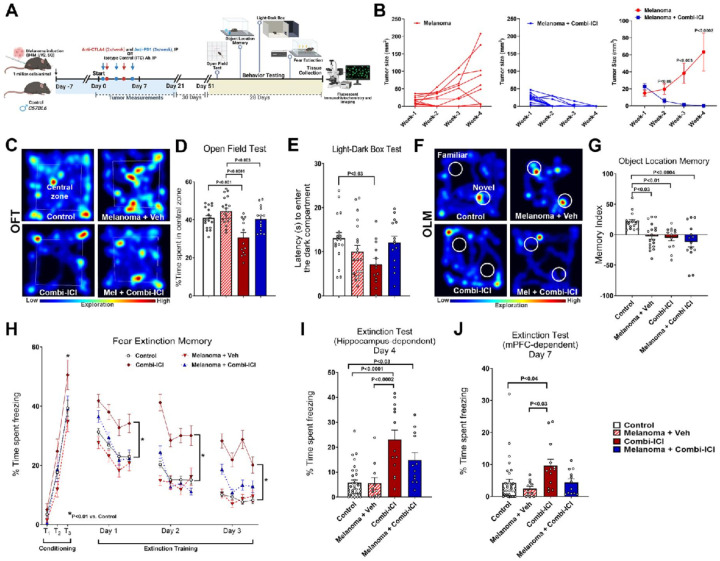
Combination blockade of CTLA-4 and PD-1 eliminates melanoma and impairs cognitive function. (A) Research design: Two-month-old C57Bl6 WT male mice received either sham or melanoma induction (D4M-UV2 cells, 1 million/animal, SQ, bilateral injections, 5 × 105 cells per site). At 7 days post-cancer induction, mice received combination ICI treatment of anti-CTLA4 (twice weekly, IP, 1000 μg) and anti-PD-1 (thrice weekly, IP, 200 μg) or vehicle for three weeks. Four weeks after the last dose, all mice were subjected to cognitive function tasks, including the Open Field Test (OFT), Light-Dark Box Test (LDB), Object Location Memory test (OLM), and Fear Extinction memory consolidation test (FE) and euthanized for tissue collection. Following groups were compared: Control (no tumor, treated with vehicle), Melanoma + Veh (tumor treated with vehicle), Combi-ICI (no tumor, treated with anti-CTLA4 & anti-PD-1), Melanoma + Combi ICI (tumor treated with anti-CTLA4 & anti-PD-1) Paraformaldehyde-fixed brains were cryosectioned to collect 30 μm thick coronal sections for immunostaining. (B) Tumor growth curves showing size of melanomas in control and combi-ICI-treated groups over four weeks, indicating a therapeutic response of combi-ICI. Plots show individual tumor growth from Melanoma and Melanoma + Combi-ICI groups and the average for each group. (C) Representative heat maps depicting open arena explored by mice during Open Field Test. (D) Percentage of time spent in the central zone (35% of the total area in C). Combi-ICI treatment reduced central zone exploration compared to corresponding controls. (E) Mice were allowed to explore an arena with a well-lit and an enclosed dark compartments during Light-Dark Box Test. Latency to enter the dark compartment (seconds) is reduced in CombiICI mice, indicative of elevated anxiety-like behavior compared to controls. (F) Mice were allowed to explore in the same arena as OFT, this time with two identical objects (block toys) placed in specific locations for OLM. Mice were then returned to the home cage. The location of one object remained the same (familiar), and the other object was placed in a new location (Novel). Five minutes later, the mice were returned to the arena. Representative heat maps depict mice exploring novel or familiar placement of objects during the OLM task. (G) Memory Index, tendency to explore novel placement of objects (from F), was calculated as: ([Novel object exploration time/Total exploration time] – [Familiar object exploration time/Total exploration time]) ×100. All treatment groups (Melanoma, combi-ICI, and melanoma + combi-ICI) showed impaired memory index compared to controls. (H) Mice were placed in an Fear Extinction testing apparatus, with a house light, a speaker, and a metal grid floor capable of delivering a shock, and a vinegar odor. During the conditioning phase (Day 0), all groups showed increased freezing following a series of three tone and shock pairings (80 dB, 0.6 mA, T1–T3); highest freezing at T3 was observed in Combi-ICI group. Each symbol represents average freezing time per group. During the extinction phase (24 h later), mice underwent fear extinction training by delivering 20 tones each day for 3 days in the same environment as the conditioning phase but without the shock. Each data point for Days 1–3 represents an average freezing time of 5 tones per mouse per group (4 data points per day). All groups showed a gradual decrease in freezing behavior (Days 1–3). Combi-ICI mice spent a significantly higher time freezing compared to the corresponding control group (*P<0.01). (I) 24 hours after the extinction training, 3 tones were played on test day in a similar environment (H) to engage the hippocampal-amygdala function. Each symbol represents an individual mouse. Higher freezing on test days shows that Combi-ICI-treated mice (irrespective of Melanoma) indicate failure to abolish Fear memory, suggesting hippocampal-dependent memory consolidation deficits. (J) 72 h after the extinction test (H & I), mice were tested in a new environment to engage the medial prefrontal cortex (mPFC). This included a white acrylic floor, additional house light, mild almond odor (10% solution in distilled water), and three tones played without shock. Each symbol represents an individual mouse. Combi-ICI group showed higher freezing compared to control and melanoma groups. All data are presented as mean ± SEM (N=12–32 mice per group). P values were derived from two-way ANOVA and Bonferroni’s multiple comparisons test.

To evaluate the effects of combination ICI (subsequently referred to as ‘combi-ICI’) on the CNS, we used a clinically relevant cancer survivor model of melanoma: syngeneic mouse model (D4M-UV2 cells) and combination therapy of CTLA-4 and PD-1 blockade in mice. D4M-UV2 is derived from D4M.3.A by ultraviolet (UV) B radiation, carries a mutational load comparable to human melanomas, is immunogenic, and responds well to combi-ICI [31]. Because the parental line D4M.3.A was initially derived from male transgenic mice, we were limited to using males in the study to avoid confounding effects of tumor rejection in female mice. As a first step, we established the efficacy of combi-ICI in our tumor model. Adult C57Bl6 WT male mice were induced melanoma using bilateral, subcutaneous injection of D4M-UV2 cells and measured for tumor growth and response to combi-ICI therapy using calipers (Fig. 1A). 10 days post-tumor induction, tumor-bearing mice were randomly enrolled into combi-ICI-treated (Melanoma-combi-ICI) or isotype control (Melanoma-Veh) groups. Combi-ICI groups received two doses of anti-CTLA-4 (clone UC10–4F10–11, BioXcell) and three doses of anti-PD1 (clone 29F.1A12^™^, BioXcell) every week for three weeks (Fig. 1A, Research Design). Age-matched control groups were injected with matching volumes and quantities of isotype control antibodies (ITC, Armenian hamster IgG, and rat IgGa). Compared to the ITC-treated mice that showed progressive melanoma growth, combi-ICI treatment significantly decreased tumor growth within a week, and by three weeks, tumor mass was undetectable (*P*<0.0002, Fig. 1B), thus confirming the therapeutic efficacy of dual ICI in our melanoma model.

After establishing a clinically relevant cancer survivor model, we next evaluated if mice treated with combi-ICI exhibited neurological symptoms similar to human counterparts. To this end, we randomly assigned mice into 4 groups: 1, Control (no tumor, treated with ITC as vehicle control); 2, Melanoma + Veh (tumor treated with ITC); 3, Combi-ICI (no tumor, treated with anti-CTLA4 & anti-PD-1); 4, Melanoma + Combi ICI (tumor treated with anti-CTLA4 & anti-PD-1) (Fig. 1A). This stratification allowed us to assess the effects of combi-ICI independent of, and in conjunction with the tumor, and potentially distinguish CNS-related effects due to tumor and combi-ICI. To determine the impact of combi-ICI treatment on learning and memory, memory consolidation, and anxiety-related tasks, four groups of mice underwent a series of cognitive function tests one month after the last dose of combi-ICI or ITC vehicle control to allow for clearance of the antibodies from their systems (Fig. 1A). These tests included open field task or activity (OFT), light-dark box test (LDB), object location memory (OLM), and, lastly, fear extinction memory task (FE). OFT and LDB measure anxiety-like behavior. OLM measures hippocampal-dependent episodic memory, and FE measures re-learning and memory consolidation processes. In the OFT, combi-ICI-treated non-cancer mice exhibited a significant reduction (*P*<0.001) in the percent time spent in the central zone compared to control mice (Fig. 1D). This open field exploration is represented in the heat map of animals from each group exploring the open arenas. Combi-ICI groups spent less time exploring the central zone compared to controls (Fig. 1C). On the LDB test, combi-ICI-treated non-cancer mice exhibited a significant decrease (*P*<0.03) in latency to enter the dark compartment compared to the control mice (Fig. 1E). Notably, these differences in OFT and LDB tests were not observed in tumor-bearing mice receiving combi-ICI, suggesting that in the presence of tumor, the anxiety-inducing behavioral effects of combi-ICI are blunted (Fig. 1D, E).

To determine whether hippocampal-dependent episodic spatial recognition memory is altered, animals were administered the OLM task (Fig. 1F–G). This task determines the animal’s ability to explore novel placement of the objects in an unrestricted, non-invasive open environment [7, 8]. This activity is calculated as a Memory Index [MI = (Novel/Total exploration time) – (Familiar/Total exploration time)] × 100]. A positive MI indicates that animals spend more time exploring novel placements of objects, indicating intact hippocampal function. In contrast, a zero or negative MI indicates that animals displayed minimal or no preference for the novel object placements and spent equal time exploring both familiar and novel places, suggesting disrupted hippocampal function. While the total time spent exploring both familiar and novel objects was comparable for each experimental group, combi-ICI-treated mice exhibited a significant decrease in the MI relative to the control (Fig. 1F, G). This decrease was also observed in melanoma-bearing mice receiving combi-ICI, suggesting that combi-ICI has a negative impact on hippocampal-dependent spatial memory. Of note, MI was also reduced in tumor-bearing mice without any therapy, likely reflecting effects of long-term tumor burden on cognitive function. This behavior is represented in the heat map of animal exploration from each for the familiar and novel placement of objects during the OLM test phase. Controls explored the novel placement more (red zones) compared to all the other groups (Fig. 1F).

Finally, to evaluate re-learning and memory consolidation processes after combi-ICI therapy, mice underwent fear extinction memory (FE) testing. All groups of mice exhibited elevated freezing after the initial tone-shock fear conditioning phase, with the most significant differences observed in combi-ICI-treated non-cancer mice (Fig. 1H, Conditioning phase, T1-T3) compared to controls (*P*<0.01) following the third tone-shock exposure. For the next three days after the 24h post-conditioning phase, mice were exposed to repeated tones without shock in the same arena with spatial (grid, house light) and odor (vinegar) cues (Fig. 1H, Extinction training phase). Mice with intact hippocampal function will exhibit a decrease in freezing behavior during tone as they re-learn during this extinction training to dissociate the previously learned freezing response following aversive stimuli (mild foot shocks). Combi-ICI-treated non-cancer mice showed elevated freezing compared to controls, indicating a decreased ability to extinguish fear. Finally, during 24-h post-extinction training, an extinction test (Fig. 1I) was administered, including exposure to tones in the same spatial and odor-cue environment as the conditioning phase (Day 1). Combi-ICI-treated mice showed a significant elevation in their freezing behavior (*P*<0.0001 and *P*<0.03 respectively vs. Control) both in the presence and absence of tumor, confirming a specific detrimental impact of Combi-ICI treatment on hippocampal-dependent fear memory consolidation process (Fig. 1I). Melanoma + Veh mice showed comparable freezing to control mice, indicating that cancer did not influence fear extinction memory. Additionally, 72 hours after the above extinction test, we assessed the frontal cortex-dependent extinction memory process in these mice. Mice were placed in a box with new flooring (acrylic plate), a spatial cue (extra light), and a new odor (almond). Throughout the 15-minute test, three tones were played without any shock. Here again, combi-ICI-treated non-cancer mice exhibited significantly higher freezing behavior than control mice (*P*<0.04). However, in this instance, the effect was not observed in the presence of the tumor (Fig. 1J). Collectively, these data demonstrate that CTLA-4 and PD-1 blockade have a selective impact on hippocampal-dependent cognitive function.

### CTLA-4 and PD-1 blockade contributes to a reduction in synaptic density and myelination

**Figure 2:**
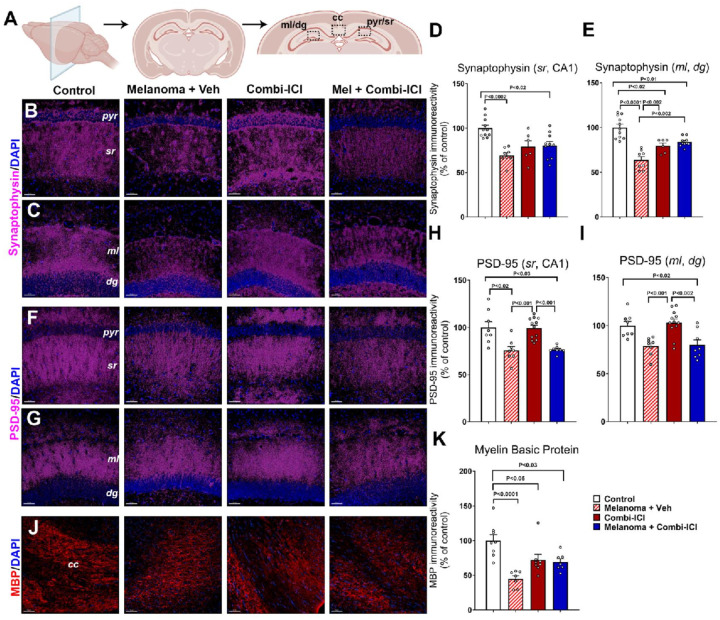
Combi-ICI treatment reduces synaptic integrity and myelination. Eight weeks after the last dose of combi-ICI treatment, mice were euthanized, their brains were fixed and collected for coronal cryosectioning, and immunostained for the pre and post-synaptic markers and myelin basic protein (MBP), workflow shown in Fig 1A. (A) Illustrations of the molecular layer (ml), dentate gyrus (dg), pyramidal neuronal layer (pyr), and stratum radiatum (sr) of the hippocampus and the corpus callosum (cc) are shown. (B-E) Representative images and volumetric quantifications showing synaptophysin immunoreactivity (magenta) and DAPI nuclear stain (blue) in the stratum radiatum (sr) layer emanating from the CA1 pyramidal (pyr) neurons (B, D) and molecular layer (ml) of the hippocampal dentate gyrus (dg, C, E). (F-I) Representative images and volumetric quantifications showing PSD-95 immunoreactivity (magenta) and DAPI nuclear stain (blue) in the ml and sr regions. (J-K) Representative images and volumetric quantifications showing MBP immunoreactivity (red) and DAPI nuclear stain (blue) in the white matter (corpus callosum, cc). All data are presented as mean ± SEM (N=8 mice per group). P values derived from ANOVA and Tukey’s post hoc test. Scale bars, 50 μm.

Cancer therapies, including cranial irradiation, have been shown to negatively impact myelination and syneptic protein levels in the brain [24]. To understand how combination ICI impairs cognitive function and contributes to neuronal damage in our melanoma model, we investigated the expression of neuronal markers that are indicators of neuronal health, including pre- and post-synaptic density proteins (synaptophysin and PSD-95, respectively, Fig. 2B–I) and myelination using myelin basic protein (MBP, Fig. 2J–K). The hippocampal molecular layer (ML) of the dentate gyrus (DG) and CA1 stratum radiatum (SR) sub-regions were analyzed for the synaptic proteins, and myelin-rich corpus callosum (CC) was analyzed for MBP (Fig. 2A). Both Melanoma + Veh and Melanoma + Combi-ICI-treated mice showed a significant decline in the hippocampal synaptophysin immunoreactivity and, therefore, pre-synaptic density in the CA1 SR (Fig. 2B, D) layers and the DG ML (Fig. 2C, E) layers. For the post-synaptic density protein, PSD-95, we found a significant decrease in the CA1 SR (Fig. 2F, H) and DG ML (Fig. 2G, I) layers in the tumor-burdened groups with or without combi-ICI treatment. These data indicate a significant synaptic loss post-cancer induction and combi-ICI treatment. Additionally, we assessed myelination in the CC using volumetric quantification of MBP immunoreactivity (Fig. 2J–K). Presence of melanoma, treatment with Combi-ICI, and Melanoma + Combi-ICI-treated groups all showed significantly lower myelination than control-vehicle animals. Taken together, these observations point to significant detrimental effects of combi-ICI treatment as well as melanoma tumor burden on synaptic and myelin integrity.

### Neuronal plasticity and neurogenesis are conserved during CTLA-4 and PD-1 blockade

**Figure 3:**
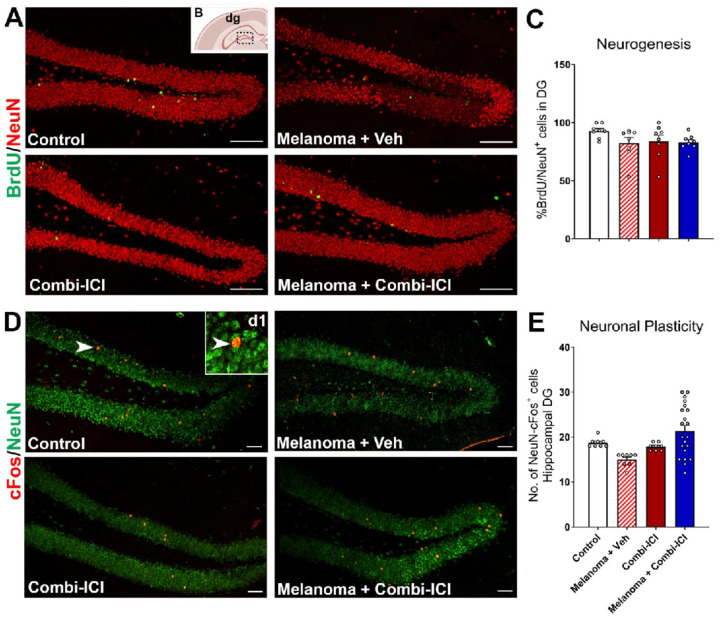
ICI treatment did not impact neurogenesis or mature neuronal plasticity. One week after the last ICI/Vehicle treatment, all mice received a thymidine analog, BrdU injections to label proliferating neural stem/progenitor cells. Brains were collected 8 weeks after the last dose of combi-CI treatment, and dual-immunostaining was conducted to determine the status of neurogenesis (BrdU-NeuN+) and neuronal plasticity (cFos-NeuN+). in the hippocampal sub-granular zone. (A-C) Representative images and quantifications of BrdU+ neural progenitor cells (Green) differentiating into mature neurons (NeuN+, Red) in the hippocampal dentate gyrus (dg, B). (D-E) Representative images and 3D quantification of a neuronal plasticity-related immediate early gene product, cFos (red), and the mature neuron marker (NeuN, green). A high-resolution confocal z stack showing dual-labeled mature neuron (NeuN) expressing cFos (d1). All data are presented as mean ± SEM (N=8–21 mice per group). P values were derived from ANOVA and Tukey’s post hoc test. Scale bars, 100 μm (A), 50 μm (D), 5 μm (d1)

Because synaptic loss can result from synaptic plasticity deficits or due to impaired neurogenesis, we evaluated the direct effects of combi-ICI on neurogenesis and neuronal activity in our tumor model. We quantified neurogenesis using BrdU-NeuN dual-immunofluorescence by estimating the percentage of mature neurons differentiated from BrdU-labeled neural stem/progenitor cells. Briefly, after the completion of three weeks of combi-ICI treatment, mice were treated with BrdU (50 mg/kg, once daily for 6 days) and euthanized about 8-weeks later. Frequencies of BrdU^+^ progenitor cells differentiating into mature neurons were comparable across all experimental groups (Fig. 3A & C), suggesting that neurogenesis is unaltered. To further determine the neuronal activity, plasticity-related immediate early gene (IEG) product cFos was co-stained with the mature neuronal marker (NeuN) in the DG blade. We found no significant differences in the number of in cFos-NeuN dual-labeled cells in the hippocampus among various groups (Fig. 3D–E). These results show that the synaptic loss associated with tumor or combi-ICI treatment is not due to effects on neurogenesis or synaptic plasticity.

### CTLA-4 and PD-1 blockade elevate microglial activation but not astrogliosis

**Figure 4:**
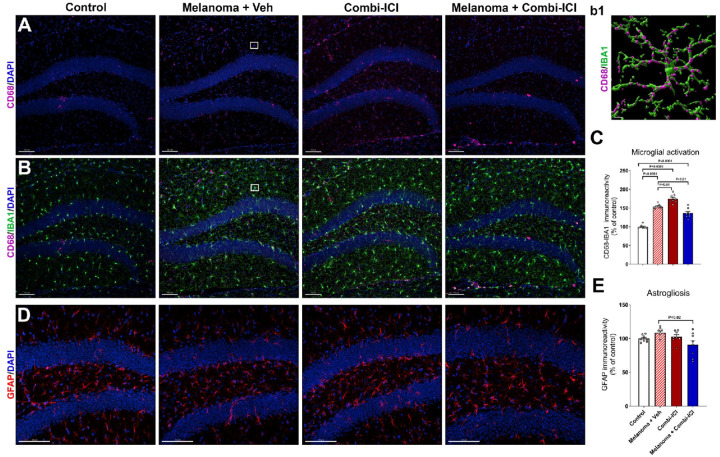
Combination ICI treatment elevates microglial activation but not astrogliosis. Eight weeks post-combi-ICI treatment, mice were euthanized, fixed brains were collected for coronal cryosectioning, and immunostained for the activated microglia and astrocyte markers (Fig 1A). (A-C) Representative images and volumetric quantification of lysosomal marker, CD68 (magenta, A) and its colocalization with the pan microglial maker, IBA1 (green, B) in the hippocampal dentate gyrus and dentate hilus (DAPI, blue, nuclear stain). A representative high-resolution surface rendering of CD68+ immunoreactive puncta (magenta) with IBA1+ microglial cell surface is shown (b1). (D-E) Representative images and volumetric quantification of astrocytic immunoreactivity (GFAP, red) and nuclear stain (DAPI, blue). All data are presented as mean ± SEM (N=8 mice per group). P values were derived from ANOVA and Tukey’s post hoc test. Scale bars, 100 μm (A, B, D), and 5 μm (b1).

We next considered the possibility that dysregulated activation of microglia and astrocytes, key players in maintaining synaptic health, could contribute to combi-ICI-mediated synaptic loss. To this end, we measured gliosis in the hippocampal dentate gyrus (DG) by assessing microglial activation and astrocytic hypertrophy status, using dual-immunofluorescence staining and laser scanning confocal microscopy at 8-weeks post-combi-ICI treatment. The quantification of CD68, IBA1 immunoreactivity, and co-localization was facilitated by *in silico* 3D algorithm-based volumetric quantification of single and co-localized surfaces as described (Fig. 4A) [34]. The combi-ICI-treated, melanoma-burdened mice treated with vehicle (Melanoma + Veh), as well as the melanoma-bearing mice receiving combi-ICI (Melanoma + combi-ICI) showed significant increases (*P*’s<0.0001) in CD68-IBA1 dual immunoreactivity in the hippocampal dentate gyrus (DG) indicating microglial activation (Fig. 4B–C). Because microglial activation is often accompanied by astrogliosis during cancer therapies [1, 34], we tested the effect of combi-ICI on glial interplay by determining the volume of GFAP immunoreactivity (astrocytes) in the DG. We did not find significant differences in GFAP immunoreactivity in any combi-ICI-treated or tumor-burdened groups compared to the control-vehicle group (Fig. 4D–E). However, we a modest decrease (*P*<0.02) was observed in the Melanoma + Combi-ICI group relative to the Melanoma + Veh (ITC)-treated group. These data suggest that combi-ICI primarily triggers microglial activation without any major effects on astrocyte reactivity.

### CTLA-4 and PD-1 blockade alters immune cells in the brain

**Figure 5:**
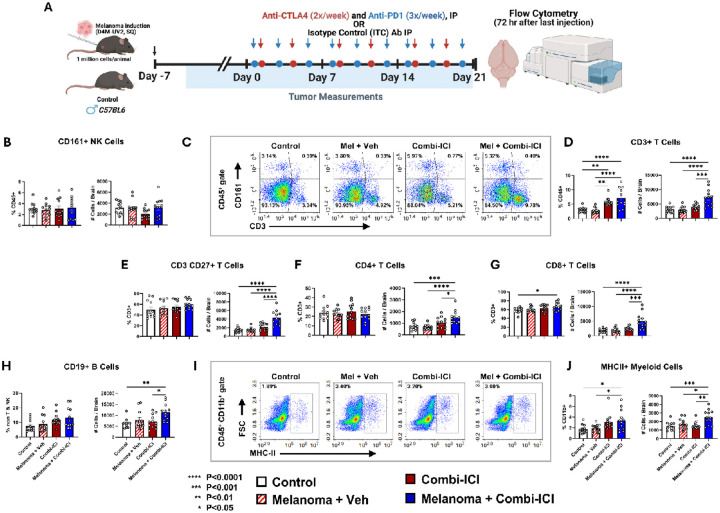
Immune cell profiling in the brain following combination ICI Treatment. (A) To immunophenotype brains using flow cytometry, mice were sacrificed at 72 hours following the last combi-ICI/Vehicle injection (B) Representative flow plots showing identification of NK cells (CD161+) and T cells (CD3 +) among immune cells (CD45+). (C) Frequencies (% of CD45) and number of NK cells (from B). There was no significant difference in the percentage or absolute number of NK cells amongst any group. (D) Frequencies (% of CD45) and number of CD3+ T cells (from B). Higher CD3 + cells in mice that received Combi-ICI (irrespective of Melanoma) indicate unleashed T cells. (E) Frequencies (% of CD3+) and total number of CD27+ CD3+ T cells (from SF2A). (F-G) Frequencies (% of CD3+) and total number of CD4+ and CD8+ T cells (from SF2B). (H) Frequencies (% of non-T & NK) and total number of CD19+ B cells (from SF2C). (I) Representative plots showing MHCII+ cells amongst CD45+ CD11b+ cells. (J) Frequencies (% of CD11b+) and number of MHCII+ CD11b+ cells (from I). All data are presented as mean ± SEM (N=12 mice per group). P values were derived from ANOVA and Tukey’s post hoc test

ICI unleashes T cells to fight cancer and also releases regulatory checkpoints on potential autoreactive immune cells in various tissues. To determine how the neuro-immune landscape changes in response to combi-ICI therapy, we used flow cytometry to comphrehensively characterize differences in immune cell subpopulations, including both lymphoid and myeloid cell populations, in brains of control and tumor-bearing mice, receiving CTLA-4 and PD-1 blockade 72 hours after the last dose of combi-ICI treatment (Fig. 5A, **Fig. S1)**. In the Melanoma-Combi-ICI group, the frequency and total number of T cells were nearly 2-fold higher than those of controls (Fig. 5D, *P’s <0.0001*). Among T cells, the frequencies of helper T cells (CD4^+^) were comparable across groups. However, there was a significantly higher frequency of cytotoxic T cells (CD8^+^) and total number of CD4 and CD8 T cells in the Melanoma-Combi-ICI group (Fig. 5E–F). Accordingly, we observed more memory T cells (CD3^+^ CD27^+^) in the Melanoma-Combi-ICI group compared to controls (Fig. 5G, **Fig S2A-B**). The number of B cells (CD19^+^) was also elevated in the Melanoma-Combi-ICI group (Fig. 5H, **Fig. S2C**).). The frequency and number of NK cells, which play a crucial role in tumor clearance, were, however, unchanged (Fig. 5B–C). Overall, these immunophenotyping results highlight a broad elevation of lymphoid cell population in the brains of cancer-bearing mice receiving combi-ICI treatment.

Among myeloid cells, Melanoma + Combi-ICI resulted in a significantly higher frequency and number of CD11b^+^ MHCII^+^ cells (Fig. 5I–J), suggesting increased antigen presentation and consistent with the findings of T cell expansion in the CNS. Melanoma alone and Melanoma + Combi-ICI groups also had elevated CD11b^+^ Ly6c^high^ cells compared to control (**Fig. S4A**), indicating infiltration of inflammatory monocytes likely driven by the tumor. On the other hand, patrolling monocytes (CD11b^+^ Ly6c^low^) that play a role in tissue repair and resolving inflammation were unaltered **(Fig. S4B)**.

Finally, cytokine levels in the plasma reflected the overall immune status following combi-ICI treatment (**Fig. S3**). Specifically, plasma levels of IL-1α, IL-6, IL-17, and TNFα, proinflammatory cytokines, were significantly higher in the Melanoma + Combi-ICI group compared to controls. Similarly, plasma IL-10, an anti-inflammatory cytokine, was also significantly higher in melanoma groups, with and without combi-ICI therapy. In contrast, plasma IL-12 (p70) level was elevated in both Combi-ICI and Melanoma + Combi-ICI, demonstrating that combi-ICI is primarily driving this change. Concurrently, we did not find statistically significant changes in tissue cytokines in the hippocampal tissues. Together with data on microglial activation and synaptic loss at a later time point, the immune phenotyping data suggest that the accumulation of a non-physiological number of lymphoid cells and the immune activation of myeloid cells during or after combi-ICI represent altered immune homeostasis in the CNS. This potentially predisposes the CNS to aberrant inflammatory responses that could dysregulate microglial function, which may drive subsequent synaptic loss.

### CTLA-4 and PD-1 blockade exacerbates CNS autoimmunity

**Figure 6:**
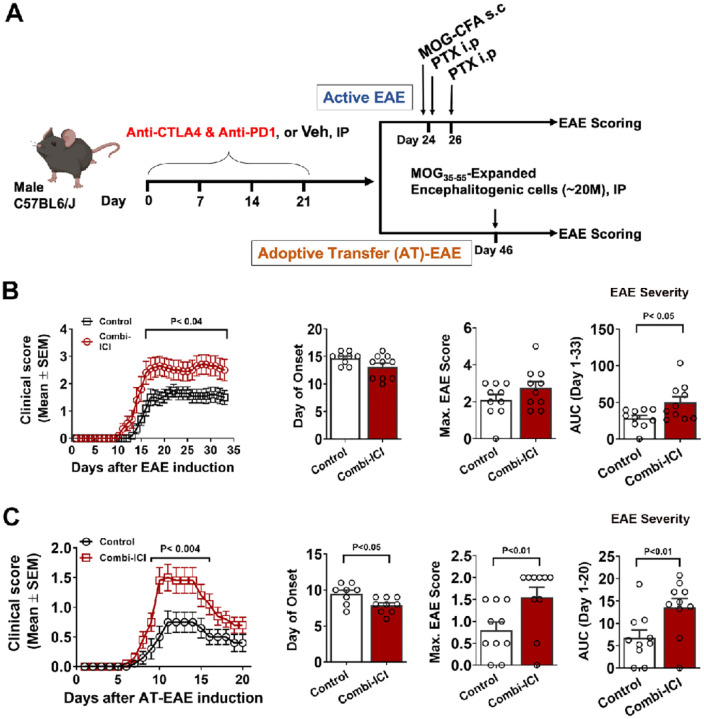
Combi-ICI predisposes CNS to heightened autoimmune encephalomyelitis. Mice underwent 3 weeks of combi-ICI treatment as described in Fig. 1A. 72 hrs after the last Combi-ICI/Vehicle injection mice were randomly enrolled into active or adoptive transfer EAE groups. (A) Experimental Design showing induction of active or adoptive transfer EAE. (B) Daily mean clinical scores, day of onset, mean maximal score, and area under the curve (AUC) analysis showing onset, progression, and overall severity of active EAE. (C) Daily mean clinical scores, day of onset, mean maximal score, and area under the curve (AUC) analysis showing onset, progression, and overall severity of adoptive transfer EAE. All data are presented as mean ± SEM (N=12–32 mice per group). P values were derived from two-way ANOVA and Bonferroni’s multiple comparisons test

To test our hypothesis that combi-ICI-mediated dysregulation of neuro-immune networks sensitizes the CNS to potential autoimmune neuroinflammatory states, we studied the effect of CTLA-4 and PD-1 blockade in experimental autoimmune encephalomyelitis (EAE), a mouse model of multiple sclerosis (MS)-like disease. Three days after combi-ICI therapy, active EAE was induced in mice with MOG_35–55_ peptide emulsified in Complete Freund’s Adjuvant (CFA) (Fig. 6A). Although disease onset was similar in both groups, the combi-ICI group displayed higher disease severity compared to the controls, measured by mean maximal score and area under the curve (~2 fold change) of EAE scores (Fig. 6B). To isolate effects of combi-ICI on the induction (activation of autoreactive T cells) and effector phase (eliciting autoimmune demyelination in the CNS) of EAE, we used an adoptive-transfer model (AT-EAE) where MOG35–55-expanded encephalitogenic cells were injected into control and combi-ICI groups [25] (Fig. 6A). The combi-ICI group now showed earlier onset and exaggerated severity (~3-fold change) of clinical scores (Fig. 6C). These results further support our hypothesis that ICI-mediated alterations of the immune homeostasis predispose the CNS to exaggerated neuroinflammation and increase the severity of autoimmunity.

## Discussion

Our study reports that melanoma and combinatorial blockade of CTLA-4 and PD-1 evoke neuroinflammatory responses (microglial activation), potentially orchestrating neurodegenerative consequences, including synaptic and myelin loss. While ICI and its combinations promote progression-free survival of individuals with cancer, cancer burden promotes certain neurodegenerative events. ICI-treated cancer survivors have increasingly reported suffering from long-term neurological complications and toxicities [17, 19, 37], which severely affects their ability to return to normalcy. However, our understanding of mechanisms is limited because it is inherently challenging to dissociate the effect of cancer from cancer therapies. Our *in vivo* mouse model identified an altered immune status sustaining neuroinflammation and neurodegeneration, underscoring the pathophysiology of ICI-related cognitive decline.

Emerging clinical observations are reporting neurological complications associated with treatment using anti-CTLA-4 and anti-PD-1 [17, 19, 37] in patients with melanoma, lung cancers, and Merkel-cell carcinoma at five weeks post-therapy [17]. Rogiers and colleagues reported long-term neurocognitive complications in about 41% of metastatic melanoma survivors up to 2 years after ICI treatment [46–48]. These clinical studies suggest that ICI treatment-induced neurotoxicity and neurocognitive decline are progressive, and clinical symptoms appear within weeks and last several years post-treatment. Our previous clinical and preclinical studies and others have shown a direct relationship between proinflammatory cytokines and CRCI following chemo- and radiation therapy [6, 11, 34, 39]. ICI-related changes in the brain functions presented in this study mirror our previous studies of chemotherapy-related cognitive impairments (hippocampal and cortex-dependent cognitive tasks) [4, 5, 12, 55], which were associated with elevated microglial activation and a significant decline in synaptic density. Elevated neuroinflammation underlies many, if not all, neurodegenerative disorders in humans, and our results support that neuroinflammation is one of the significant contributory factors for long-term CRCI. Limiting neuroinflammation to mitigate ICI-related cognitive impairments is a potential avenue to advance cancer immunotherapeutics further.

### Combinatorial ICI-related cognitive dysfunction.

Melanoma-bearing and control mice receiving combinatorial ICI treatment underwent behavior and cognitive function testing two months post-treatment. The open field test (OFT) and anxiety-related light-dark box (LDB) test did not show increased anxiety in cancer-bearing mice (Fig. 1C–E), indicating an absence of neophobic behavior during cognitive function testing. ICI-treated (no-cancer) mice showed reduced time spent in the open zone (OFT) and reduced latency in entering the dark compartment (LDB). Thus, ICI-treated mice were less explorative due to a marginal increase in anxiety. This resulted in them spending more time in the peripheral zones of the open arena (OFT) or the dark compartment (LDB). Subsequently, mice were tested on a hippocampal-dependent object location memory (OLM) task that determined spatial recognition memory (Fig. 1F–G). The presence of cancer itself (Melanoma + Vehicle) and ICI treatment in either cancer or no-cancer mice led to a significant decline in the performance of the OLM task, indicating disruption of hippocampal function. Lastly, mice were conditioned and trained on fear extinction and tested for the hippocampal- and medial prefrontal cortex (mPFC)-dependent fear extinction memory tests (Fig.1H–J). During the conditioning phase (three tone-shock pairs), all groups of mice showed progressively increased freezing. Mice without cancer and receiving ICI treatment showed the highest freezing compared to controls (P<0.01). This behavior is related to their increased anxiety levels, as observed in the OFT and LDB tasks, and reflects an elevated response to the aversive stimuli (tone-shock exposure). For the next three days, all groups of mice received fear extinction training (repeated tones without shocks) in the same spatial context and the odor cue 24 hours apart (Fig. 1H). All groups of mice showed decreased freezing, except for ICI-treated mice without cancer, which remained frozen and did not learn to extinguish the aversive memory, indicating their inability to relearn. The spatial context (arena walls, grid flooring, and the odor cue, vinegar) remained unchanged while the animals underwent extinction training. Intact hippocampal function is essential for retrieving spatial information. Thus, elevated freezing during extinction training further indicates hippocampal-selective deficits in the Combi-ICI-exposed mice. At 24 hours post-extinction training, ICI treatment to cancer-bearing or non-cancer mice during the extinction test resulted in a significantly elevated freezing response. These mice were unable to extinguish fear memory despite three days of training in the same arena, indicating a compromised hippocampal-dependent fear memory-consolidation or relearning process (Fig. 1I). Melanoma-bearing mice receiving the vehicle did not differ from the controls. At 24 hours after the extinction test, the context (acrylic flooring, additional house light) and the odor cue (almond) within the fear testing arena were changed to engage mPFC. In this test, no-cancer mice receiving the combination of ICI remain frozen compared to controls (Fig. 1J). In summary, melanoma and combination ICI impaired episodic memory (OLM); however, combination ICI, in the presence and absence of melanoma, significantly disrupted higher memory consolidation processes, leading to long-term cognitive dysfunction.

### Neuroinflammation-related loss of myelin and synaptic integrity.

We evaluated surrogate markers of neuronal health, including myelin (MBP), pre-and post-synaptic density proteins (synaptophysin and PSD-95, respectively), and neuronal plasticity IEG marker, cFos-NeuN. Mice without cancer and receiving combination ICI, cancer-bearing mice with or without ICI treatment showed a significant decline in the myelin basic protein immunoreactivity (Fig. 2). Both altred numbers of immune cells and resident immune cell (microglia) activation may lead to myelin damage. Our results from the active model of EAE suggest that ICI pretreatment exposes CNS to exaggerated CNS autoimmunity (Fig. 6). During MOG_35–55_-EAE, infiltration and activation of CNS-reactive CD4 T cells and other cells, including CD8 T cells, NK cells, and B lymphocytes, orchestrate demyelination and axonal damage [40]. To rule out the effect of combination ICI on the peripheral immune system during active EAE, we used an adoptive transfer EAE model. Accordingly, we also observed similar results of exaggerated EAE, strengthening our hypothesis that an ICI-mediated neuroinflammation-like state is conducive for potential autoreactive lymphocytes to orchestrate higher myelin damage levels. These results explain the mechanistic basis of relapsing disease, including mortality observed in MS patients after ICI therapy for various cancers [21].

After combinatorial ICI treatment, we found elevated expression of MHC class II positive cells and CD68-IBA1 positive activated microglia in the brain (Fig. 4 & 5). Chronic neuroinflammation has been linked with long-term myelin loss and progressive neurodegeneration *in vivo* [32]. Indeed, we found a loss of synaptic integrity in the hippocampal molecular layer (ML) and CA1 stratum radiatum of the dentate gyrus (DG), which is important for the learning and memory processes. Combination-ICI treatment to intact or melanoma-bearing mice and melanoma burden itself led to a significant decline in the pre-synaptic protein, synaptophysin, at two months post-ICI treatment (Fig. 2). Synaptic loss was more pronounced in melanoma-bearing mice receiving vehicle or ICI treatments for the post-synaptic density protein PSD-95 (Fig. 2). Synaptophysin plays an essential role in pre-synaptic vesicular trafficking, and its loss is often associated with neurodegenerative conditions, including Alzheimer’s disease [18, 22]. We have also shown reduced synaptophysin levels in the irradiated brains coincident with cognitive decline [34, 43]. Post-synaptic density protein, PSD-95, is a scaffolding protein that plays essential roles in excitatory neurotransmitter receptor clustering and function [22, 27]. Combi-ICI treatment-induced loss of myelin and synaptic proteins could inadvertently impact neuronal health and neurotransmission. We found no change in expression of an immediate early gene (IEG), cFos, within the mature neurons (NeuN^+^) following ICI treatment (Fig. 3). Additionally, hippocampal neurogenesis (BrdU-NeuN), the formation of new neurons from neural stem/progenitor cells, was unchanged (Fig. 3). While mature neuron differentiation and neuronal plasticity are critical functions, they are not the sole hallmarks of impaired neuronal health. Collectively, combi-ICI- and inflammatory environment-induced myelin and pre- and post-synaptic protein loss indicate a long-term detrimental effect on the neuron functional landscape that could culminate into neurocognitive impairments.

### Microglial activation post-ICI treatment.

Combinatorial ICI therapy using anti-CTLA4 and anti-PD1 controlled melanoma growth *in vivo* (Fig. 1B). This model allowed us to study the impact of such targeted cancer therapy on brain function. We found that melanoma induction and combination ICI treatment to non-cancer and cancer-bearing mice led to a significant increase in microglial activation (Fig. 4), as shown by elevated hippocampal dual-immunoreactivity for the CD68 and IBA1 at two months post-combination ICI treatments. An increased microglial activation in melanoma-bearing mice (~150%) indicates chronic cancer burden itself. A modest reduction of CD68-IBA1 volume in ICI-responsive cancer mice compared to melanoma indicates tumor therapeutic response. We did not find a significant increase in astrogliosis *in vivo* (Fig. 4). IBA1 is a pan microglial marker expressed by both resting and activated microglia, whereas CD68 is a lysosomal protein marker expressed by phagocytotic microglia and macrophages. Long-term elevation in the CD68-IBA1 dual-immunoreactivity following cancer induction and ICI treatment indicates proinflammatory (phagocytotic) microglia activation that could harm neuronal and synaptic health. Our past studies using chemotherapy and radiation therapy in rodent models have shown elevated neuroinflammation, particularly microglial activation, in the brain, leading to cognitive impairments [1–5, 34, 36]. Microglia are essential in clearing dead cells, sub-cellular debris, and synaptic pruning *in vivo* [41, 42]. Proinflammatory activation of microglia may lead to excessive synaptic pruning and neuronal damage, leading to compromised neuronal and neurocognitive function.

### Combination ICI alters neuro-immune homeostasis.

Elevated cytokine levels, including IL1α, TNFα, IL6, IL10, IL17, and IL12, represent the rejuvenation of tumor immunity in response to combination ICI. Our assessment of immune cells in the CNS using flow cytometry provides key insights into the cellular mechanisms following the combination of ICI treatment of melanoma. A higher frequency of MHCII-positive myeloid cells and T cells indicates a Combi-ICI-mediated neuroinflammation-like state in the CNS. ICI antibodies have been reported to enter CNS [56, 58], which could directly act on their targets in the CNS resident immune and non-immune cells. Elevated T, including memory phenotype (CD27+), particularly in the cancer + treatment group, could be driven by the following possibilities, which need in-depth investigation. *i) CTLA-4 and T cells*: Blockade of CTLA-4 unleashes T cells and expands T cell receptor (TCR) repertoire in the periphery [38, 44, 45, 52], some of these αCTLA4-unleashed (CNS antigen-reactive) T cells reach the brain and initiate a cascade of autoimmune neuroinflammation and orchestrate neurodegeneration; *ii) Tissue T cells*: in addition to unleashing T cells in the periphery, PD1 blockade unleashes tissue-resident memory T (T_RM_) cells which usually express high levels of PD1 [13, 33, 51], and these αPD1-unleashed Tcells drive autoimmune damage to the CNS [20, 57] and dysregulate neuro-immune homeostasis. *iii) PD1 and microglia*: PD1 blockade disrupts homeostatic glial PD1 (microglia)-PDL-1 (astrocytes) axis [28, 30] resulting in microglial activation [58]; and *iv) Combi-ICI and emergent phenomenon*: Combined blockade of CTLA-4 and PD1 synergically [60, 61] unleashes T cells, and in conjunction with disruption of PD1-PDL1 axis in the brain, activates microglia, thus potentiating local inflammatory loops that sustain abberent synaptic pruunning and myelin damage.

To what extent CLTA-4 and PD1 monotherapies alter the neuro-immune landscape compared to Combi-ICI is an important question. A recent study has demonstrated that PD-1 monotherapy (e.g., B16 melanoma and MC38 colon cancer models) directly activate microglia, and ablating microglia was sufficient to rescue αPD-1-mediated cognitive deficits [58]. Our results strengthen these findings and establish that selectively limiting microglial activation could be key to mitigating immune-related adverse events (irAE) in the CNS. Because CTLA-4 is primarily expressed by T cells, additional conditional knockout studies could resolve the relative contribution of T cells and microglia during Combi-ICI-mediated neuroinflammation and neurodegeneration. The effect of ICI and their combinations on innate lymphoid-like cells (ILCs) needs further investigation. Future studies identifying microglia-specific immune regulatory networks and regional variations (e.g., cortex, leptomeninges) could reveal novel molecular targets to decouple undesired neurotoxicities associated with ICI therapies while maintaining anti-tumor responses.

## Conclusions

To summarize, reactive microglia, synaptic loss, diminished myelin, higher number of inflammatory monocytes in the brain, and elevated plasma IL-10 in melanoma-bearing mice indicate cancer burden-associated changes compared to healthy control. Melanoma-bearing mice performed similarly to healthy controls in most cognitive function tests except object location memory. Combi-ICI treatment alone activated microglia, affected synaptic integrity, damaged myelin, increased CNS T cells, predisposed CNS to exaggerated autoimmunity, and significantly disrupted cognitive function. Combi-ICI treatment of melanoma recapitulated individual effects of the tumor as well as therapy modeling clinical scenario. Primarily, elevated plasma cytokines reflect tumor control and a pronounced increase in CNS T cells indicates Combi-ICI mediated unleashing of potentially autoreactive T cells.

The immune system constantly protects the CNS and plays a critical role in development, homeostasis, and preserving cognitive function. Dysregulated neuro-immune interactions are associated with neurodegenerative conditions and cognitive decline; our results demonstrate key neuroinflammatory events associated with Combi-ICI treatment. Our preclinical observations of significantly diminished hippocampal-dependent cognitive functions in melanoma-bearing mice treated with combi-ICI are highly relevant for quality-of-life issues among cancer survivors. Recent studies report neurocognitive sequelae of ICI in more than 40% of cancer survivors [17, 35, 46, 47]. Our earlier studies identified similar neuroinflammation-related cognitive impairments during chemotherapy- and cranial radiation therapy-exposed brains [1–5, 34, 36]. Our current study highlights the role of neuroinflammation-mediated neurodegenerative consequences of Combi-ICI. Based on the foregoing, our working model is that cancer treatment using Combi-ICI alters the immune landscape in the brain, snowballing into neuronal and synaptic damage, leading to cognitive dysfunction. Thus, mitigation strategies to thwart or reduce ICI-mediated neuroinflammation, including microglial activation, without compromising anti-cancer efficacy need careful consideration to prevent potential cognitive decline and improve the overall quality of life for thousands of cancer survivors.

## Data Availability

All data and materials are available upon reasonable request.

## ABBREVIATIONS

ANOVA: Analysis of Variance
Anti-CTLA-4: Cytotoxic T-Lymphocyte-associated Protein 4 Antibody
Anti-PD-1: Programmed Cell Death Protein 1 Antibody
AT: AT Adoptive Transfer
BrdU: 5-Bromo-2′-deoxyuridine
CA1: Cornu Ammonis 1
CC: Corpus Callosum
CD: Cluster of Differentiation
CFA: Complete Freund’s Adjuvant
CNS: Central Nervous System
Combi-ICI: Combination Immune Checkpoint Inhibition
CRCI: Cancer-Related Cognitive Impairments
CTLA-4: Cytotoxic T-Lymphocyte-Associated Protein 4
DG: Dentate Gyrus
e.g.: Exempli Gratia
EAE: Experimental Autoimmune Encephalomyelitis
FACS: Fluorescence-Activated Cell Sorting
FDA: Food & Drug Administration
FE: Fear Extinction
GFAP: Glial Fibrillary Acidic Protein
IBA1: Ionized Calcium-Binding Adaptor Molecule 1
ICI: Immune Checkpoint Inhibitors
IEG: Immediate Early Gene
Ig: Immunoglobulin
IL: Interleukin
ILCs: Innate Lymphoid-like Cells
irAEs: Immune-Related Adverse Events
ITC: Isotype Control
LDB: Light Dark Box
Ly6c: Lymphocyte Antigen 6 Complex
MBP: Myelin Basic Protein
MHCII: Major Histocompatibility Complex II
MI: Memory Index
ML: Molecular Layer
MOG_35–55_: Myelin Oligodendrocyte Glycoprotein Peptide 35–55
mPFC: Medial Prefontal Cortex
MS: Multiple Sclerosis
NeuN: Neuronal Nuclear Protein
NK: Natural Killer Cells
NSCLC: Non-Small Cell Lung Cancer
OFT: Open Field Test
OLM: Object Location Memory
P: Probability Value
PD-1: Programmed Cell Death Protein 1
PSD-95: Post-Synaptic Density Protein 95
PTSD: Post-Traumatic Stress Disorder
SEM: Standard Error Mean
SR: Stratum Radiatum
T1-T3: Tone 1-Tone 3
TCR: T Cell Receptor
TNFα: Tumor Necrosis Factor Alpha
T_RM_: Tissue-Resident Memory T cells
US: United States
UV: Ultraviolet
Veh: Vehicle
WT: Wildtype
αCTLA: Cytotoxic T-Lymphocyte-associated Protein 4 Antibody
αPD-1: Programmed Cell Death Protein 1 Antibody
